# The role of CD180 in hematological malignancies and inflammatory disorders

**DOI:** 10.1186/s10020-023-00682-x

**Published:** 2023-07-17

**Authors:** Kurtis Edwards, Peter M. Lydyard, Nino Kulikova, Tamar Tsertsvadze, Emanuela V. Volpi, Nicholas Chiorazzi, Nino Porakishvili

**Affiliations:** 1 School of Life Sciences, University of Westminster, London, UK; 2 The University of Georgia, Tbilisi, Georgia; 3 Division of Infection of Immunity, University College London, Gower Street, London, WC1E 6BT UK; 4 Agricultural University of Georgia, Tbilisi, Georgia; 5 Javakhishvili Tbilisi State University, Tbilisi, Georgia; 6 The Feinstein Institute, New York, NY USA

**Keywords:** CD180, Toll-like receptors, Chronic lymphocytic leukemia, Innate immunity, Inflammation, Systemic lupus erythematosus, Hematological malignancies

## Abstract

Toll-like receptors play a significant role in the innate immune system and are also involved in the pathophysiology of many different diseases. Over the past 35 years, there have been a growing number of publications exploring the role of the orphan toll-like receptor, CD180. We therefore set out to provide a narrative review of the current evidence surrounding CD180 in both health and disease. We first explore the evidence surrounding the role of CD180 in physiology including its expression, function and signaling in antigen presenting cells (APCs) (dendritic cells, monocytes, and B cells). We particularly focus on the role of CD180 as a modulator of other TLRs including TLR2, TLR4, and TLR9. We then discuss the role of CD180 in inflammatory and autoimmune diseases, as well as in hematological malignancies of B cell origin, including chronic lymphocytic leukemia (CLL). Based on this evidence we produce a current model for CD180 in disease and explore the potential role for CD180 as both a prognostic biomarker and therapeutic target. Throughout, we highlight specific areas of research which should be addressed to further the understanding of CD180 biology and the translational potential of research into CD180 in various diseases.

## Introduction

Research into the innate immune system has undergone a renaissance in recent times with a growing appreciation of the importance of this immunological system outside of its classical ‘innate’ functions. This has been hallmarked by an ever-increasing interest towards innate immune receptor expression and function. Perhaps most notably, toll-like receptors (TLRs) are gaining traction as important immunological players which regulate various physiological functions and play a role in many different diseases, including cancer and inflammatory disorders. Over the past 35 years, there have been a growing number of publications on the expression and function of the orphan TLR, CD180, with significant literature exploring the role of the receptor in disease.

CD180 was initially discovered on human B cells through reactivity with a mouse monoclonal antibody (clone G28.8) prepared for one of the annual International Human Leukocyte Antigen workshops in the 1980s (Valentine et al. 1988). The same group also showed that the binding of the G28.8 antibody to its target induced activation of human B cells (Valentine et al. 1988). The target antigen, identified later on murine B cells, was characterized as a radioprotective molecule of approximately 105 kDa (RP105) (Miyake et al. 1995) and was eventually dubbed CD180 in the new cluster of differentiation (CD) nomenclature. Earlier studies using transfection to isolate the RP105/CD180 antigen detected the presence of a leucine rich repeat (LRR) motif, which was the first indication that RP105 could be homologous with the TLR family (Miyake et al. 1995). Later, sequencing data, coupled with phylogenetic analysis, confirmed that CD180 had a high homology with TLRs, especially TLR4, and it was therefore included into the TLR family (Miura et al. 1996; Divanovic et al. 2005; Fugier-Vivier et al. 1997). Further studies showed that CD180 is present on a number of cell types, other than B lymphocytes, including dendritic cells (DCs) and macrophages (Divanovic et al. 2005) and has several distinct physiological roles.

More recently, CD180 was shown to be present on malignant hematological cells. Functional studies using primary cells and immortalized cell lines have indicated that CD180 plays a significant role in the immunopathology of these cancers. Various publications on autoimmune and inflammatory disorders have also implicated CD180 in the pathophysiology of these diseases.

In this review, we discuss the characteristics and function(s) of CD180 in both normal physiology and pathological conditions and compare features of CD180 to other TLRs. We discuss the role of CD180 in hematological malignancies, autoimmune diseases, and other inflammatory diseases. In addition, we highlight how this knowledge may translate into further research and/or practice in the future.

## The molecular structure of CD180

**Fig. 1:**
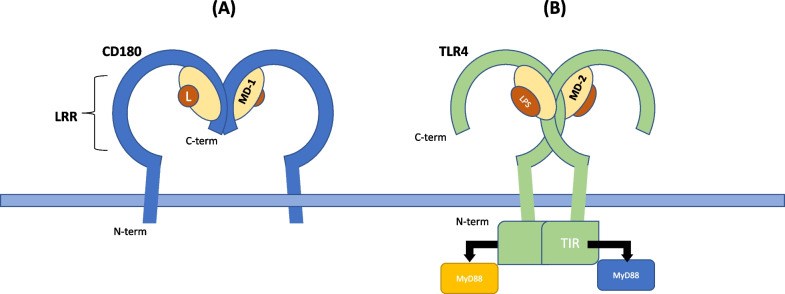
The structure of CD180 compared to TLR4. The extracellular domain of CD180/MD-1 contains leucine rich repeats (LRR) and dimerizes at the C-terminus, thus forming a head-to-head topology (**A**), which contrasts TLR4/MD-2 that dimerizes at the N-terminus (**B**). CD180/MD-1 complex forms a 2:2 heterodimer, perhaps in the presence of a lipid molecule (L), which acts as a ligand and binds to the hydrophobic cavity of MD-1. This is analogous to TLR4/MD-2 which binds to lipopolysaccharide (LPS) via a lipid-binding cavity found within MD-2. Whilst CD180 has a small intracellular portion, the intracellular portion of TLR4/MD-2 contains a toll-like/interleukin-1 receptor (TIR) box domain which enables the initiation of intracellular signaling via two alternative pathways: myeloid differentiation primary-response protein 88 (MyD88)-dependent and MyD88-independent

In humans and mice, some TLRs including CD180 are found on the cell surface, whereas others are intracellular and located within endosomes (Medzhitov 2001). CD180 is a type 1 single-pass transmembrane protein with a molecular mass of 105 kDa and is made up of 661 amino acids in humans (Fugier-Vivier et al. 1997) and 641 in mice (Miyake et al. 1995); the amino acid sequence shares a high degree of sequence and structural homology with other TLRs (Miyake et al. 1995). CD180’s extracellular LRR motif (Takeda et al. 2003) forms a horseshoe-like topology (Miura et al. 1996; Ohto et al. 2011) which is typical of TLRs (Fig. 1). CD180 exhibits strong similarity with TLR4 (Divanovic et al. 2005) and it forms a 2:2 heterodimer with myeloid differentiation 1 (MD-1) at its LRR motif (Ohto et al. 2011; Yoon et al. 2011). The association of CD180 with MD-1 is essential for its surface membrane expression in mice and humans (Nagai et al. 2002; Miyake et al. 1998; Miura et al. 1998). Similarly, TLR4 is also a heterodimer and requires myeloid differentiation 2 (MD-2) for stable membrane expression (Visintin et al. 2006).

TLR4 and CD180 also differ in their intracellular domains. All classical TLRs, including TLR4, contain a toll-like/interleukin-1 receptor (TIR) domain which enables signal propagation preferentially via myeloid differentiation primary-response protein 88 (MyD88)- or toll/interleukin-1 receptor domain-containing adaptor-inducing interferon-β (TRIF)-dependent pathways (Kawasaki and Kawai 2014; Miyake 2004). However, CD180 does not have a TIR domain but instead possesses a very short intracytoplasmic tail (Miyake et al. 1995) (Fig. 1). Thus, CD180 cannot recruit the typical TLR-associated intracellular signaling components (Yazawa et al. 2003).

## Potential ligands for CD180

CD180 is referred to as an ‘orphan’ TLR since its natural ligands have not yet been identified. The other TLRs have a wide variety of ligands ranging from flagellin to DNA and RNA of microbes (Kawasaki and Kawai 2014). These microbial-associated ligands are categorized as microbe-associated molecular patterns (MAMPs)—formerly called pathogen-associated molecular patterns (PAMPs)—and their recognition is a pivotal part of innate immune responses. The TLR4/MD-2 complex detects lipopolysaccharide (LPS) from Gram-negative bacteria through the lipid A binding pocket found within MD-2 (Fig. 1); however, early research indicated that LPS did not bind to MD-1 (Tsuneyoshi et al. 2005). Though, this was subsequently challenged by crystallographic studies which demonstrated direct LPS interactions with MD-1 via a lipid-like moiety (Ohto et al. 2011; Yoon et al. 2010) (Fig. 1), suggesting the CD180/MD-1 complex also can directly bind and respond to LPS (see Function of CD180). However, LPS is unlikely to be a CD180 ligand, but rather is sensed in tandem with TLR4, since TLR4/MD-2 CD180/MD1-negative human embryonic kidney cells (HEK) 293 cells are unresponsive to LPS (Liu et al. 2013).

Crystallographic studies have been utilized to investigate how MD-1 may bind to lipid molecules, such as lipid IVa in chickens (Yoon et al. 2010) and phosphatidylcholine in mice (Harada et al. 2010). Using mass spectrometry, Ohto, Miyake and Shimizu later also determined electron densities which were consistent with the presence of lipid molecules bound to the MD-1 portion of the murine CD180/MD-1 complex (Ohto et al. 2011). In accordance with the conservation in structure between CD180 and TLR4, the evidence to date suggests that the ligand for CD180 is likely to bind to the MD-1 portion of the complex, as is the case with TLR4/MD-2 and LPS (Fig. 1). Schultz et al. also showed that synthetic lipopeptide molecules, that mimicked the lipoproteins expressed by *Mycobacterium tuberculosis* and *Mycobacterium bovis*, resulted in CD180-dependent activation of macrophages (Schultz et al. 2018). Therefore, *Mycobacterium*-derived MAMPs may be natural CD180 ligands.

In addition to MAMPs, many endogenous danger molecules (damage-associated molecular patterns, DAMPs) are released upon tissue damage, cell death and oxidative stress. These molecules include the phospholipid OxPAPC, high mobility group Box 1 (HMGB1), S100 proteins, heat shock proteins, and many more that are also sensed by TLRs (Yu et al. 2010). It is therefore possible that CD180 has both natural microbe-associated and endogenous ligands that are yet to be discovered.

Whilst both the endogenous and exogenous ligands for CD180 are yet to be established, it is possible that CD180 detects its ligands through homodimerization (Ohto et al. 2011; Yoon et al. 2011) (see section on CD180 structure). CD180 might also heterodimerize with other TLRs, thereby allowing interactions with ligands through well characterized mechanisms. For example, TLR1/2 and TLR2/6 form heterodimers to detect lipoproteins/lipopeptides, and TLR1/10, TLR2/TLR10, and TLR4/TLR6 can heterodimerize to detect endogenous DAMPs. CD180 is known to physically associate with TLR4 and TLR2 (Divanovic et al. 2005; Nagai et al. 2005) and, therefore, these TLRs are candidates which could form heterodimers with CD180 for ligand detection. Moreover, a model by which CD180 heterodimerizes with other TLRs could explain how CD180 initiates signaling events, despite its short intracytoplasmic tail. The search for CD180 ligands is ongoing and will be crucial for developing our understanding of CD180 in health and disease.

## CD180 in different cell populations: expression, signaling and function

CD180 is readily expressed by cells of the haematopoietic system, in particular antigen presenting cells: DCs, macrophages and B cells (Zarember and Godowski 2002). Thus, the human spleen and lymph nodes are the anatomical sites with the greatest expression of CD180 (along with other TLRs) (Zarember and Godowski 2002; Roshak et al. 1999). However, since CD180, unlike several of the TLRs, is not expressed by epithelial cells lining the digestive (Testro and Visvanathan 2009) and urinary tracts (Samuelsson et al. 2004), and the uterus (Schaefer et al. 2004), it is assumed that CD180 does not provide the classical ‘sentinel’ function of TLRs at entry points into the body.

CD180 is expressed at the highest levels on both human (Valentine et al. 1988) and murine mature B cells (Miyake et al. 1995), although certain heterogeneity exists in the expression of CD180 amongst some defined B cell subsets. Murine follicular (FO) B cells exhibit a reduced expression of CD180 compared to marginal zone (MZ) B cells (Nagai et al. 2005). Furthermore, based on immunohistochemistry studies of normal human tonsils, our group demonstrated that CD180 is highly expressed within the marginal zone, but not in the germinal centres (Edwards et al. 2021), a pattern which is mirrored in murine lymph node tissues (Miura et al. 1998). The expression of CD180 was also found to be around two-fold higher by memory B cells compared to naïve B cells (Good et al. 2009).

In humans, there appears to be no difference in the expression of CD180 in the CD5^+ ^B cell subset compared with the CD5^−^ subset in the peripheral blood (Porakishvili et al. 2005). Furthermore, fully differentiated human B cells (plasma cells) do not normally express CD180 (Kikuchi et al. 2018).

Early data indicated that some molecules found on the surface of B cells might regulate expression of CD180. Activation of human B cells with anti-IgM stimulating antibodies decreases the expression of CD180 (Porakishvili et al. 2005), suggesting that activated B cells express less CD180 than resting B cells. Studies of murine B cells also showed that ligation of TLR7 or TLR9 can downregulate the expression of CD180 (You et al. 2017). Further, the expression of CD180 on B cells can differ, and tends to be downregulated, in malignant, autoimmune, and inflammatory pathologies.

### CD180-mediated B cell signaling and activation

Signaling through most TLRs results in activation through TIRAP (toll/interleukin 1-receptor adaptor protein) and MyD88 leading to induction of pro-inflammatory cytokines. TLR4 also has another signaling pathway mediated by the adaptor proteins TRIF and TRAM (TRIF related adaptor molecule), leading to production of type 1 interferons. Although CD180 lacks a TIR domain, it possesses a short intracellular portion of around 11 amino acids. It is possible that CD180 interacts with the B cell receptor (BCR) through this tail.

The exact CD180 signaling pathway(s) is/are still unclear in normal B cells (Fig. 3). Signaling events in knockout mice (KO) and human B cells showed that CD180 ligation leads to the phosphorylation of various intracellular protein kinases including Syk, Lyn, p38MAPK, ERK and AKT (Porakishvili et al. 2011; Chan et al. 1998). Whilst these intracellular signaling patterns are reminiscent of BCR signaling, CD180 activation fails to effectively mobilize intracellular calcium as IgM engagement does (Porakishvili et al. 2011; Chan et al. 1998).

**Fig. 2:**
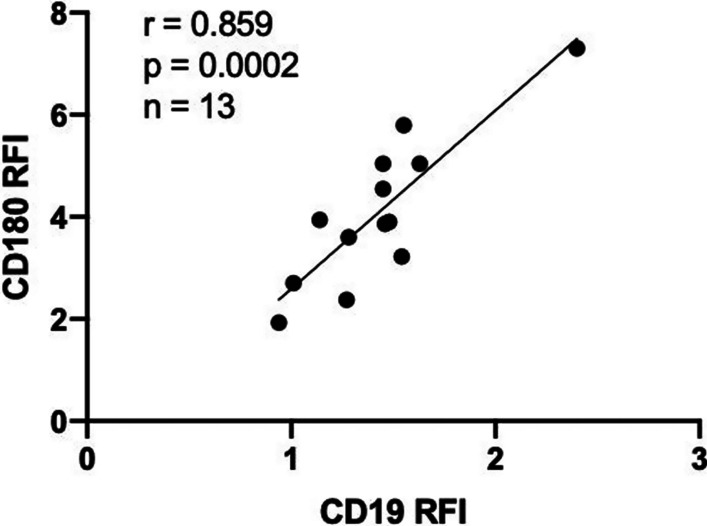
The relationship between CD180 and CD19 in normal B cells. Using Pearson’s correlation, the density of CD180 correlated strongly with CD19 on normal B cells. PBMC samples from healthy donors were stained with anti-CD180 (PE) and anti-CD19 (FITC) monoclonal antibodies (mAb). Antigen densities were measured by flow cytometry and expressed as a ratio to the fluorescence of the isotype control—relative fluorescent intensity (RFI)

CD19 is important in CD180 signaling, based on evidence in CD19 mouse KO experiments and on the fact that ligation of CD180 leads to CD19 phosphorylation; CD19 deficiency therefore leads to hyporesponsiveness to CD180 ligation (Yazawa et al. 2003). It is also noteworthy that we have observed strong correlations between the density of CD180 and CD19 in normal B cells (Fig. 2). Importantly, CD19 may serve as an adaptor molecule which facilitates the recruitment of Lyn and Vav upon CD180 activation (Yazawa et al. 2003).

Lyn and Vav phosphorylation are important early signaling events in CD19 activation (Sato et al. 1997); thus, CD180 may activate a similar signaling program. Dependency of murine B cells on Lyn signaling has been evidenced in KO mice studies. Lyn^−/−^ mice showed an attenuated activation of MAPK isoforms compared to wild-type mice, indicating a significant role of Lyn in early CD180-mediated signal transduction (Chan et al. 1998).

**Fig. 3:**
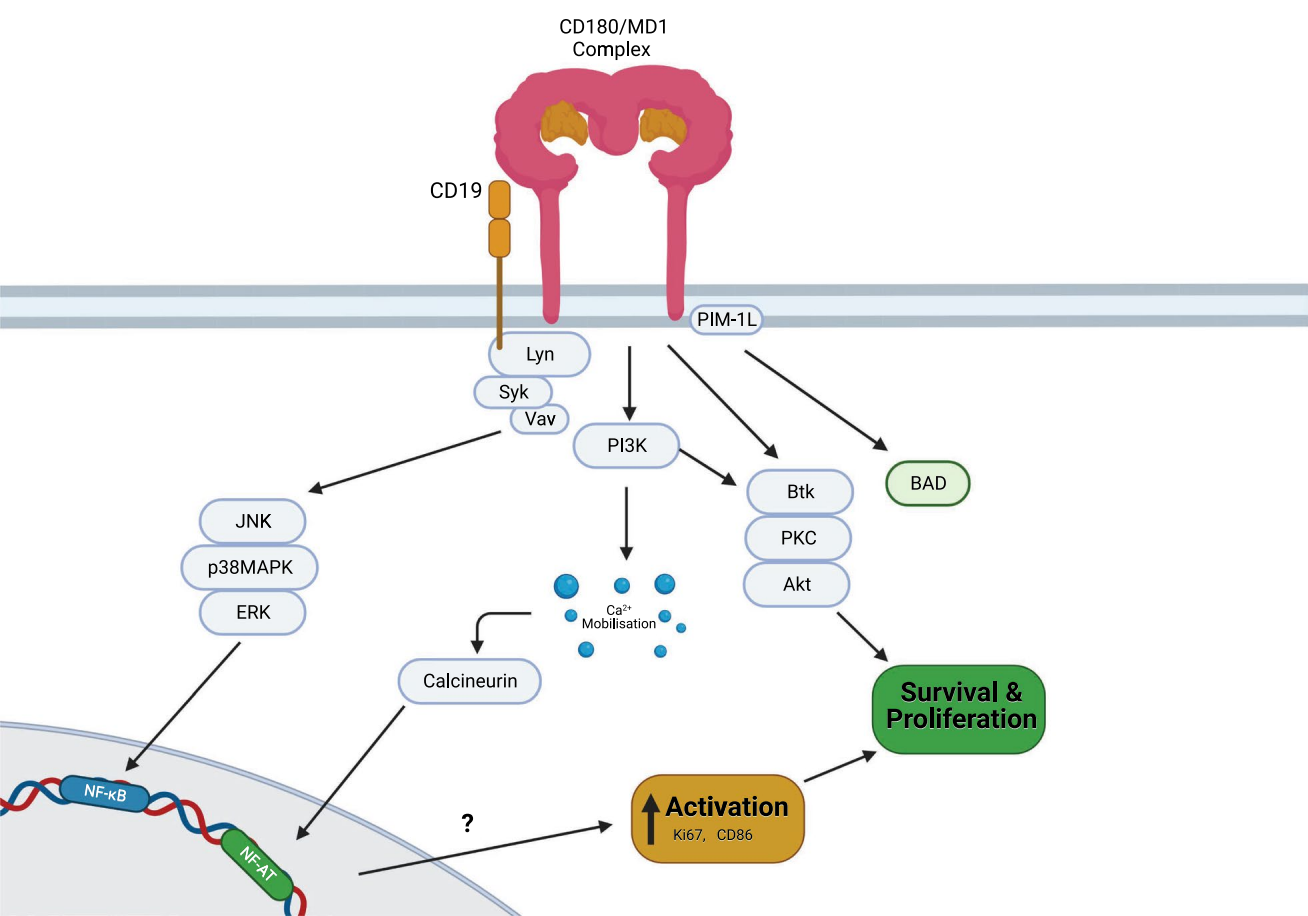
CD180 signaling in human B cells. Upon CD180 engagement with anti-CD180, CD19 becomes phosphorylated and associates with CD180 in the membrane. Downstream signaling members Vav appear to be crucial for transducing CD180 signals. CD180 concomitantly activates mitogen activated protein kinases and Akt signaling in healthy B cells, both of which appear to contribute to the upregulation of costimulatory molecules and cell survival. CD180 also associates with the PIM-1L kinase at the cell surface, which, upon activation of CD180 with anti-CD180, results in increasing levels of BAD. B cells can also receive signals from CD40 and interleukin 4 (IL-4) receptors to promote proliferation and survival whilst CD40 activation may promote upregulation of PIM-1L

PIM-1L-kinase is also phosphorylated upon ligation of CD180 on human B cells and increases the phosphorylation of the anti-apoptotic protein BAD. PIM-L1 kinase also co-localizes, and is physically associated with CD180 (Egli et al. 2015), implying that it is an early adaptor molecule in CD180 signal transduction (Fig. 3**)**.

Following ligation of CD180 (with murine monoclonal antibody to CD180—mAb) in healthy B cells, both the AKT and mitogen activated protein (MAP) kinase pathways become activated, ultimately converging into the upregulation of the Nuclear factor kappa-light-chain-enhancer of activated B cells (NF-kB) pathway (Yazawa et al. 2003; You et al. 2017) (Fig. 3). In turn, healthy B cells upregulate expression of the activation and proliferation markers CD86 and Ki-67 (Porakishvili et al. 2005, 2011) and increase their production of immunoglobulins (Chaplin et al. 2011). More detailed signaling studies, especially with identified ligands, will be important to link the early signaling events to downstream players and eventually to transcription factors other than NF-kB.

### Murine B cells

Some of the earliest functional studies on CD180 demonstrated that this receptor is related to B cell activation. Ligation of CD180 on murine B cells (Roshak et al. 1999) led to cell activation, proliferation, and protection from radiation and dexamethasone-induced apoptosis (Miyake et al. 1995; Miura et al. 1998). In 2005, Nagai et al. first demonstrated additional CD180 functions. Iinjection of mice with anti-CD180 upregulated transcription factors associated with plasma cell differentiation and increased the number of transitional and mature B cells but did not affect memory B cells (Nagai et al. 2005). It also increased production of IgG1 and IgG3, but not IgG2b or IgA (Chaplin et al. 2011). Further, coupling an antigen (Ag) to CD180 antibodies increased IgG production by B cells, even in CD40^−/−^ and T cell-deficient mice (Chaplin et al. 2013). Antigen targeting to CD180 also increased the number of antigen-specific B cells, compared to the targeting of CD40, which resulted in the proliferation of antigen non-specific B cells (Chaplin et al. 2013). Data from these experiments, therefore indicated a synergy between CD180 and the BCR for effective antigen responses. 

The physiological function of CD180 is still unclear, and it is likely that resolution will ultimately require identification of its in vivo ligand(s). As described above, in mice, LPS seems to bind to the CD180/MD-1 complex, but the role of LPS in generating CD180-dependent cell signaling is still debatable. There are, however, data suggesting that CD180 on B cells regulates the function/expression of some of the classical TLRs.

#### CD180 regulates TLR2 and TLR4 function

Unlike TLR4 and other TLRs, CD180 ligation alone does not lead to cytokine production (Chaplin et al. 2011); instead, CD180 regulates cytokine production through modulation of TLR function in murine B cells. In 2000, Ogata et al. first showed that CD180-deficient B cells exhibit hyporesponsiveness to LPS (Ogata et al. 2000). It was later shown that wild type murine B cells responded to LPS or lipid A with anti-CD180 mAb by upregulation of CD86, whereas B cells from MD-1^−/−^ mice had an attenuated response (Nagai et al. 2005). Interestingly, CD180^−/−^ mice also had reduced Ig production in response to LPS compared to wild type (WT) mice. Later, Yazawa et al. also showed that, by using antibodies to block MD-1, LPS-mediated phosphorylation of CD19 was completely inhibited. This further indicated the importance of CD180/MD-1 in the regulation of LPS detection by murine B cells (Yazawa et al. 2003).

The exact role which CD180 plays in the regulation of TLR-mediated LPS signaling in mice has, however, been challenged. Allen et al. showed that, whilst B cells from CD180^−/−^ mice have a reduced proliferative response to LPS, the mice remain responsive to other proliferative stimuli (Allen et al. 2012). Moreover, MZ and FO B cells from CD180^−/−^ mice show comparable upregulation of CD69 and MHC II as well as no differences in the production of IL-10 or IL-6 in response to LPS challenge. Consistently, the same study also found that basal B cell proliferation was increased, especially within the MZ B cell subset. Together, these findings suggest that defective responses to LPS by murine B cells may not be completely explained by the CD180^−/−^ genotype, as suggested by Allen et al.

B cell activating factor (BAFF) is a key survival factor for B cells and is also important in TLR signaling. CD180^−/−^ mice show an elevated expression of BAFF by qPCR, and neutralisation of BAFF reversed both the LPS hyporesponsive phenotype and elevated basal proliferation (Allen et al. 2012), implying a role of BAFF in CD180-mediated LPS responses. Whilst the data from KO mice studies indicate a role of CD180 in LPS responsiveness, the findings by Allen et al. (2012) cannot be ignored. They suggest that absence of CD180 might not be the only reason for attenuation of murine B cell LPS responsiveness but rather that disrupted expression/function of other factors related to CD180 activation may be at the root of defective LPS detection within this genotype. Exploration of other signaling players which function downstream of CD180 will enable more accurate evaluation of how CD180 disruption contributes to LPS hyporesponsiveness in murine B cells.

Like TLR4, CD180 appears to exert regulatory influence on TLR2 in murine B cells. CD180^−/−^ mice have poor proliferative responses, diminished plasma cell differentiation, and defective polyclonal Ig production in response to TLR2 (but not TLR9) ligands (Nagai et al. 2005), suggesting a dichotomous role of CD180 in B cell proliferation and modulation of TLR responses. B cells from CD180^−/−^ mice also show an attenuated upregulation of CD69 and CD86 in response to synthetic lipopeptides compared to wild type (WT) controls, which further implicates CD180 in the modulation of TLR2 function (Blumenthal et al. 2009). Unlike TLR-mediated cytokine production in murine B cells, ligation of CD180 alone fails to induce cytokine production, yet, together with TLR2 and TLR4 ligands, CD180 engagement increases production of IL-6, IL-10 and tumor necrosis factor alpha (TNFα) (Chaplin et al. 2011).

CD180 in murine B cells, therefore, appears to be involved in enhancement/modulation of specific cytokine function through TLR2 and TLR4, as well as inducing proliferation, activation, and antibody production.

### Human B cells

Antibody-mediated stimulation of CD180 on human peripheral blood B cells activates a programme of intracellular signaling and leads to an increase in the expression of activation markers and proliferation (Porakishvili et al. 2005, 2011). Direct stimulation through CD180 also leads to increased cell survival, perhaps in part via anti-apoptotic proteins such as BAD (Egli et al. 2015) or activation of NF-kB in an AKT-dependent manner (Yamazaki et al. 2010; Porakishvili et al. 2015). Therefore, the function of CD180 on human B cells is directed primarily towards cell activation, survival, proliferation, and antibody production.

#### CD180 regulates the function of TLR9 in human B cells

Yamazaki et al. (2010) showed that TLR9 expression within the endosomes increased following treatment of both naïve and memory B cells with anti-CD180 antibodies. The same authors also demonstrated that treatment with anti-CD180 and the TLR9 ligand CpG drastically increased the proportion of viable B naïve cells in culture, compared to naïve B cells treated with CpG alone. Moreover, the effect of the engagement of CD180 plus TLR9 was synergistic, increasing the phosphorylation of AKT. However, stimulation of B cells with anti-CD180 alone failed to significantly induce AKT phosphorylation. These results are discordant with our own studies in normal human B cells, since our group (Porakishvili et al. 2015) and another (Gordiienko et al. 2017a) have found that CD180 activation induced significant phosphorylation of AKT (and p38MAPK).

#### CD180 and TLR9 regulate antibody production

CpG activation of TLR9 serves as a stimulus to promote polyclonal Ig production following infection (Bernasconi et al. 2199). Yamazaki et al. found that CpG treatment increased the production of IgA, IgD, and IgM (Yamazaki et al. 2010). The same authors also showed that anti-CD180 antibodies did not enhance Ig production in the presence of anti-CD40, CpG, or SAC (*Staphylococcus aureus* Cowan strain 1) or IL-21 treatment. These findings suggest that production of immunoglobulins by human and murine B cells diverges in its response to the CD180 ligation.

Interestingly, the vitamin A metabolite, *all trans* retinoic acid (ATRA), a nutrient significantly correlated with immunological health as well as B cell function (Blomhoff et al. 1992; Ertesvag et al. 2007), may help to regulate the Ig production via CD180 and TLR9. Eriksen et al. reported that ATRA treatment of both naïve and memory B cells increased the proliferative effects of anti-CD180 treatment alone and together with the TLR9 ligand CpG (Eriksen et al. 2012). Further, ATRA treatment attenuated the CD180-dependent production of IgG. However, it also drastically enhanced IgG production in memory B cells synergistically activated with anti-CD180 and CpG. Consistently, B cells stimulated with both, anti-CD180 and CpG, significantly increased production of IL-10 following ATRA treatment, whereas ATRA almost completely abrogated the production of IL-10 in B cells treated with anti-CD180 alone. Together, these findings suggest that TLR9, CD180, and ATRA work in concert for optimum antibody production. Moreover, the fact that CD180 activation alone leads to a dampening of memory B cell activation could indicate that CD180 serves as a negative regulator to suppress excessive immune responses. This concept aligns with the studies of CD180 in autoimmune disease where loss of CD180 tends to be associated with a more severe disease phenotype (see section on CD180 in disease).

B cell proliferation and Ig production by the proposed “CD180/TLR9 axis” appears to be regulated, at least partially, by reactive oxygen species (ROS). By-products of mitochondrial respiration act as second messengers in intracellular signaling systems, including BCR signaling (Tsubata 2020). It was shown recently that CD180/TLR9-mediated production of Ig was accompanied by the activation of NADPH oxidase (NOX) (Gilljam et al. 2020). N-acetyl cystine (NAC), a ROS scavenger, also selectively reduced the production of IgG, rather than IgM, initiated by CD180/TLR9 activation (Gilljam et al. 2020). Therefore, the respiratory function, mitochondrial health, and production of ROS in B cells may also regulate isotype-specific Ig production and proliferation via combined CD180 and TL9 signaling.

Building on the work investigating the role of ATRA in B cell function with respect to CD180 and TLR9, Eriksen et al. investigated the role of ATRA on CD180 and TLR9-mediated autophagy (Eriksen et al. 2015), since autophagy is a process apparently involved in the regulation of murine B cell differentiation and Ig production (Arnold et al. 2016). The addition of ATRA to B cells stimulated with anti-CD180 and CpG was found to significantly increase the formation of autophagosomes in both naïve and memory B cells. Moreover, B cells treated with ATRA, CpG, and anti-CD180 mAb induced autophagosome and lysosome colocalization, as well as the number of autophagosomes, indicating a role for these three stimuli in the remodelling and self-digestion of B cells. The production of IgG was also dependent on the transcription of the Unc-51 Like Autophagy Activating Kinase 1 (ULK1), which is increased significantly by stimulating cells with ATRA, CpG, and anti-CD180 together (Eriksen et al. 2015). Therefore, in combination, CD180 and TLR9 function to regulate ATRA-mediated autophagy of B cells, which may be important for effective Ig responses.

Together, the data accrued so far implicate a significant role for CD180 in the biology of B cells. CD180 has stand-alone functions, whereby it can promote activation and proliferation of B cells as well as production of immunoglobulins. Further, CD180 works in concert with classical members of the TLR family, apparently exerting regulatory function over immune activation in response to microenvironmental stimuli. Differences in CD180 function also exist between mice and man, particularly with respect to TLR function, given that murine B cells and human B cells differ in their TLR expression profiles. Furthermore, CD180 may play a role in modulating the function of other TLRs expressed on B cells and indeed other classes of receptors.

### Myeloid cells

CD180 is highly expressed on both murine and human monocytes and macrophages (Zarember and Godowski 2002; Roshak et al. 1999), but not granulocytes (Tsertsvadze et al. 2015). Myeloid DCs express CD180 and TLR4 (Divanovic et al. 2005), whereas plasmacytoid DCs only appear to express CD180 intracellularly (Schuster et al. 2010) and do not produce TLR4 (Krug et al. 2001). The CD180/MD-1 complex has also been shown to be physically associated with both TLR4/MD-2 and with TLR2 in immunofluorescence and immunoprecipitation studies with murine macrophages and TLR expression models using human embryonic kidney (HEK) 293 cells (Divanovic et al. 2005; Blumenthal et al. 2009).

#### Regulation of TLR function

CD180 is an activation molecule on B cells and is involved in antibody production, cell activation, and modulation of TLR signaling. CD180-deficent murine DCs and macrophages show greater responsiveness to LPS (in terms of cytokine production) compared to their WT counterparts (Divanovic et al. 2005), indicating a role of CD180 in the negative regulation of TLR4. Consistently, murine macrophages, pre-treated with blocking anti-MD-1 and then stimulated with LPS, produced more TNFα and IL-12-p40 but less IL-10 and chemokine ligand 5 (CCL-5) compared to unstimulated or isotype control-stimulated cells (Liu et al. 2013).

Experimental model systems utilizing the human HEK293 cell line to study TLR signaling also support the negative role of CD180 on TLR4 in myeloid cells. HEK293 cells, transfected with cDNA in order to express the CD180/MD-1 and TLR4/MD-2 complexes, show reduced IL-8 production in response to LPS challenge, compared to cells expressing TLR4/MD-2 alone (Divanovic et al. 2005). CD180 is also expressed by murine myeloid derived suppressor cells (MDSCs) and can negatively regulate responses to endotoxin via TLR4 (Dong et al. 2019).

In contrast, Nagai et al. showed that knockout of CD180 did not impair the production of TNFα in response to TLR4 or TLR2 ligands in either murine DCs or macrophages (Nagai et al. 2005). This report was consistent with a later study which also found that macrophages from CD180^−/−^ mice did not have attenuated cytokine responses following treatment with TLR2 or TLR4 ligands (Blumenthal et al. 2009).

The apparently conflicting data on the CD180-mediated regulation of TLR2 or TLR4 may be explained by concentrations/availability of the respective ligands. Liu et al. found that cytokine responses in murine macrophages, pre-treated with blocking anti-MD-1 antibodies and then concomitantly treated with the TLR2 ligand Pam3CSK4, and TLR4 ligand LPS, were similar to controls (Liu et al. 2010). However, when the dosage of Pam3CSK4 was increased, production of pro-inflammatory cytokines was significantly enhanced. This is consistent with previous findings where TLR2 activation was found to overcome CD180-dependent negative regulation of TLR4-mediated LPS detection (Divanovic et al. 2005).

It is notable that HEK293 cells expressing only the CD180/MD-1 complex do not produce cytokines in response to LPS or Pam3CSK4, indicating that these ligands cannot directly induce signaling via CD180. Furthermore, murine macrophages and DCs, but not B cells, respond poorly to anti-CD180 mAbs (Divanovic et al. 2005; Nagai et al. 2005). Therefore, the data accrued so far suggest that CD180/MD-1 regulates the function of TLR4 and TLR2 in macrophages and DCs rather than acting as a lone activation molecule, and this differs from B cells, which robustly respond to anti-CD180 (Porakishvili et al. 2005, 2011).

#### Regulation of antigen presentation

The role of CD180 in myeloid cells has also been explored within the context of antigen processing. In 2013, Chaplin, Chappell and Clark showed that mice with CD180-sufficent B cells, but CD180-deficient non-B cells, produced significantly less IgG compared to mice where CD180 was expressed in both B cells and non-B-cells (Chaplin et al. 2013). This suggested that CD180 expression by APCs from both the myeloid and lymphoid lineages is required for optimum antibody response. The fact that DCs, treated with antigen-coupled anti-CD180, were able to more robustly stimulate the proliferation of antigen-specific T cells, compared to B cells also lends credence to this concept (Chaplin et al. 2013; Roe et al. 2019). Further, antigen targeting of CD180 also improved the capacity of DCs to stimulate antigen-specific T helper cells compared to targeting of CD40 (Roe et al. 2019). Therefore, CD180 functions to promote effective antibody responses through both DCs and B cells.

## The expression of CD180 in B lymphocyte malignancies

The expression of CD180 on B cells has led to the study of the molecule in B lymphocyte malignancies where CD180 expression and function appears to diverge from its normal B cell counterparts. CD180 expression and function have also been studied in other non-malignant pathologies, such as autoimmune and inflammatory pathologies, and these will be addressed in the next section. There is now a growing body of evidence implicating CD180 in disease, and this research has begun to highlight how CD180 could be exploited as a diagnostic, a prognostic, or even a therapeutic target.

### Heterogeneity of CD180 expression in leukemias and lymphomas

CD180 appears to be mainly confined to malignancies of the B cell lineage (Valentine et al. 1988; Miyake et al. 1995; Divanovic et al. 2005; Zarember and Godowski 2002; Porakishvili et al. 2005) with only one report on acute myeloid leukemia which demonstrated that *CD180* gene expression is high in leukemic stem cells (Saito et al. 2010).

CD180 and other TLRs are variably expressed in malignancies of B cell origin. In both leukemia and lymphomas, the patterns of CD180 expression are heterogenous. Using flow cytometry, it was shown that CD180 was weakly expressed by malignant cells in chronic lymphocytic leukemia (CLL), mantle cell lymphoma and lymphoplasmacytic lymphoma, compared with control  B cells (Miguet et al. 2013; Mayeur-Rousse et al. 2016). The level of expression of CD180 in hairy cell leukemia, marginal zone lymphoma (MZL), follicular lymphoma (FL) and splenic diffuse red pulp small B cell lymphoma was found to be comparable to that of control B cells (Miguet et al. 2013; Mayeur-Rousse et al. 2016; Mestrallet et al. 2016). Interestingly, FL cells isolated from lymph nodes showed higher expression of CD180 compared to FL cells isolated from the peripheral blood, whereas CD180 expression was comparable in the different tissue compartments in MZL, MCL and CLL (Mestrallet et al. 2016).

A multicentre study suggested that the high density of CD180 in marginal zone lymphoma (MZL) can be used in a test panel as a marker to distinguish MZL from other lymphomas and leukemias with similar characteristics (Mayeur-Rousse et al. 2016). Other combinations of markers used in conjunction with CD180, such as CD148 and CD200 (Miguet et al. 2015) or CD148 alone (Gautam et al. 2021), have been suggested to be useful for distinguishing MZL from other malignancies such as mantle cell lymphoma (MCL). These marker combinations could be especially helpful for determining a differential diagnosis in malignant B cell neoplasms when there are atypical presentations of immunophenotypic markers (Gautam et al. 2021). Notably, CD43 and CD180 have recently been explored as markers which can be used to diagnose atypical CLL (Li et al. 2021). This combination may be particularly useful in Asian populations where atypical immunophenotypic presentations are more common (Tomomatsu et al. 2010).

Table 1 shows the relative expression of CD180 in B cell leukemias and lymphomas in the peripheral blood.

**Table 1:** The expression of CD180 in leukemias and lymphomas

Malignancy	Expression	References
Normal B cells	+ + + +	(Valentine et al. 1988; Porakishvili et al. 2005)
Chronic lymphocytic leukemia	+	(Porakishvili et al. 2005; Miguet et al. 2013; Mayeur-Rousse et al. 2016; Mestrallet et al. 2016)
Follicular lymphoma (lymph node)	+ + +	(Mayeur-Rousse et al. 2016; Mestrallet et al. 2016)
Follicular lymphoma (peripheral blood)	+ +	(Mayeur-Rousse et al. 2016; Mestrallet et al. 2016)
Hairy cell leukemia	+ + +	(Mayeur-Rousse et al. 2016)
Mantle zell lymphoma	+	(Miguet et al. 2013; Mayeur-Rousse et al. 2016; Mestrallet et al. 2016)
Marginal zone lymphoma (splenic)	+ + + +	(Miguet et al. 2013)
Marginal zone lymphoma (nodal)	+ +	(Miguet et al. 2013)
Splenic diffuse red pulp small B cell lymphoma	+ + + +	(Miguet et al. 2013)

### Chronic lymphocytic leukemia

Chronic lymphocytic leukemia (CLL) is probably the most studied of the hematological malignancies with regard to the evaluation of the expression and function of CD180. Around 60% of CLL cases express CD180, with the range of expression density varying as a continuum. However, even in CLL cases where CD180 is considered to be highly expressed, its density is lower than that on normal B cells (Porakishvili et al. 2005; Arvaniti et al. 2011). In the majority of CLL cases where CD180 was not expressed on the cell surface, the protein was found within the cytoplasm (Gordiienko et al. 2017a) (and unpublished data).

#### CD180 signaling in CLL

As for normal B cells, studies on ligation of CD180 through anti-CD180 showed upregulation of CD86 and MHC class II and Ki67 (Porakishvili et al. 2011, 2004). Interestingly, only 50% of CD180-positive CLL samples showed this effect (responder samples), and this group of cases included both CLL cells with mutated *IGVH* genes (M-CLL) and unmutated *IGVH* genes (U-CLL). Importantly, it was established that those CD180-positive but unresponsive M-CLL and U-CLL samples also responded poorly to the ligation of CD40 or the addition of recombinant IL-4 (rIL-4) (Porakishvili et al. 2011). Thus, unresponsiveness to CD180 ligation might delineate CLL cell anergy.

**Fig. 4:**
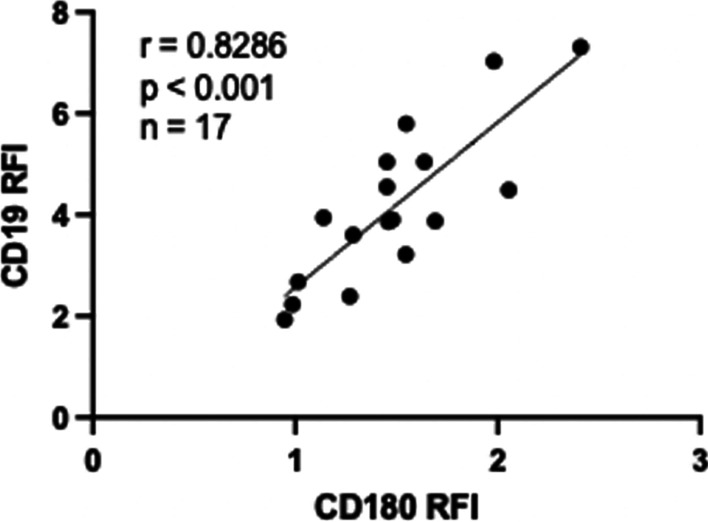
The relationship between CD180 and CD19 expression on CLL cells. Using Pearson’s correlation, the density of CD180 and CD19 expression was found to be highly correlated on CLL cells. PBMC samples from CLL donors were stained with anti-human CD180 (PE) and anti-human CD19 (FITC). Antigen densities were measured by flow cytometry and expressed as a ratio to the isotype control. Relative fluorescent intensity (RFI)

Ligation of CD180 on CD180-positive responder CLL samples resulted in cell activation, cell cycling, and phosphorylation of intracellular protein kinases (Porakishvili et al. 2011). The level of cell activation achieved by CD180 ligation was also found to be equivalent, or in some cases superior to the activation achieved with anti-CD40 or rIL-4 (Porakishvili et al. 2011), which are known to robustly induce CLL cell activation and protect from apoptosis (Falco et al. 2018). This occurs despite CD180-positive samples expressing much less CD180 compared to normal B cells, underscoring the potent signaling capacity of CD180 in CLL cells. Moreover, the combined ligation of CD180 and CD40, along with rIL-4 treatment, results in significantly increased activation and cell cycling (Porakishvili et al. 2005, 2011). These data suggest that CD180 can be independently but synergistically activated alongside CD40 and IL-4 receptor (Chaplin et al. 2011). Should these pathways cooperate together in vivo*,* this may result in increased CLL cell activation and tumor burden expansion. As previously mentioned, CD19 is also a major regulator of CD180 signaling, becoming phosphorylated and forming a complex with Vav and Lyn following CD180 activation (Yazawa et al. 2003). We have also found that the expression of CD180 and CD19 are highly correlated on CLL cells (Fig. 4).

**Fig. 5:**
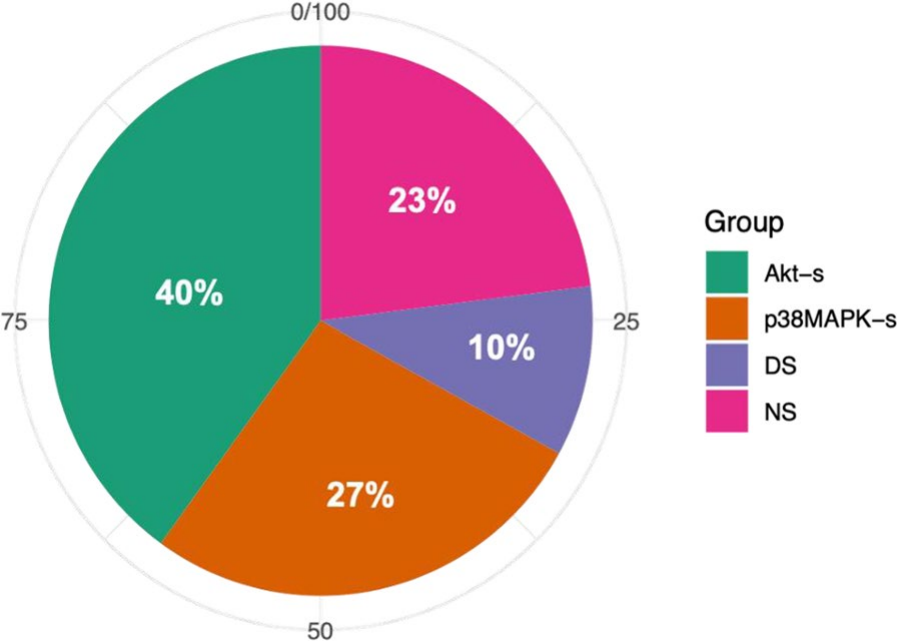
Categorization of CLL cells into signaler type. CLL cells can be categorized into four distinct groups depending on the phosphorylation (p) patterns of intracellular protein kinases p-AKT and p-p38MAPK following treatment with anti-CD180 mAb. double-signaler (DS), non-signaler (NS)

As described above, CD180 ligation on normal B cells leads to simultaneous signaling through AKT and p38MAPK pathways. However, most individual CLL samples use one of these signaling pathways, with a minority engaging both or neither (Porakishvili et al. 2015). Patterns of expression are shown in Fig. 5 based on their capacity to increase phosphorylated (p) p-AKT (AKT-signalers, AKT-S) and p-p38MAPK (p38MAPK-signalers or p38MAPK-S) above basal levels. Importantly, in our hands, upon CD180 ligation AKT-S and p38MAPK-S CLL samples reduce phosphorylation of the alternative protein kinases to below basal levels, indicating that this reduction might condition the signaling dichotomy observed in CLL.

Studies on larger cohorts of CLL patients are required to validate the proportions of patients categorized within these signaling groups at diagnosis and provide information as to whether treatment can affect CD180 signaling dichotomy.

Importantly, AKT-S samples signal for survival, whilst p38MAPK-S samples tend to undergo apoptosis upon CD180 ligation (Porakishvili et al. 2015). This is consistent with the fact that activation of the AKT axis is pro-survival in CLL (Chapman et al. 2017; Zhuang et al. 2010) and thus, AKT-S could represent a compartment of CLL cases with more aggressive disease. However, the pathophysiological role of p38MAPK in CLL is unclear. Support for a role of p38MAPK in a pro-apoptotic pathway comes from studies showing that apoptosis caused by novel cell cycle inhibitors (Paiva et al. 2015) and immunosuppressive agents (Bai et al. 2011) is mediated by p38MAPK. In addition, CD180 overexpression in cardiomyocytes can induce apoptosis via p38MAPK phosphorylation in ischemia/reperfusion injury rat models (Li et al. 2016; Yang et al. 2015).

The dichotomous signaling pattern in CLL seems to be an acquired feature, and it is unknown if this pattern is observed in other B cell malignancies. The role of this dichotomy in CLL is presently unclear but does not appear to relate to surface density of CD180 or the co-expression of IgM, IgD or CD38 (Porakishvili et al. 2015). One of the putative kinases downstream from p38MAPK could include JNK1/2 and mTORc1, since CD180 ligation activates mTORc1 and JNK1/2 (Gordiienko et al. 2017a). Moreover, cyclin-dependent kinase inhibitor P1446A induces apoptosis of CLL cells via JNK/p38 (Paiva et al. 2015). It is possible that CD180 ligation could induce a similar chain of signaling events resulting in apoptosis.

#### CD180 and the B cell receptor (IgM)

Signaling through the BCR of B cells and CLL cells occurs mainly via the AKT pathway leading to proliferation and survival. Studies have shown that cross-talk between TLRs and the BCR can promote pro-survival signaling in lymph node-resident CLL cells (Dadashian et al. 2019; Herishanu et al. 2011). CD180 and the BCR can also interact in mouse B cells and in CLL cells to modify intracellular signaling.

**Fig. 6:**
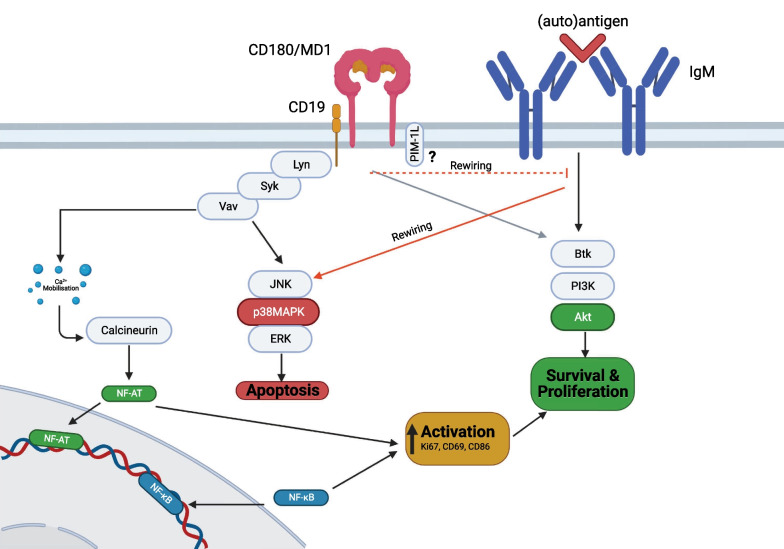
CD180 signaling in CLL. CD180 can signal dichotomously via a pro-survival BTK/PI3K/AKT pathway or via a pro-apoptotic pathway mediated by p38MAPK, which is accompanied by a downregulation of AKT. Activation of the AKT axis, which is thought to also involve activation of PI3K, mediates an increase in anti-apoptotic Mcl-1 and BCL-_XL_ activity; and is also coupled with a downregulation in p38MAPK. Pre-engagement of CD180 can inhibit AKT-mediated signaling via IgM (dashed line), which tends to be accompanied by an increase in the phosphorylation of p38MAPK and the number of apoptotic CLL cells. Co-ligation of both CD150 and CD180 together attenuates both of AKT and p38MAPK pathway signaling

Pre-engagement of CD180 followed by sIgM ligation caused AKT-S samples to rewire signals from a pro-survival (AKT-S) to a pro-apoptotic pathway (p38MAPk-S) (Porakishvili et al. 2015) (Fig. 6). Similarly, in murine B cells, pre-treatment with anti-CD180 antibodies resulted in apoptosis following subsequent BCR activation (Yamashita et al. 1996). This cross-talk could also be amplified by IL-4, which increases the expression of IgM and enhances its function in CLL cells (Aguilar-Hernandez et al. 2016). IL-4 can be produced by CLL cells in an autocrine fashion or by T cells (Kay et al. 2001).

#### CD180 and CD150

CD150 is part of the signaling lymphocyte activation molecule family (SLAMF1). It positively correlates with the expression of CD180 in CLL, and around 59% of cases express both CD150 and CD180 (Gordiienko et al. 2017a), consistent with independent studies (Porakishvili et al. 2005, 2011; Bologna et al. 2016) and our own unpublished observations. Moreover, CD150 and CD180 colocalize together in the surface membrane with CD19 (Gordiienko et al. 2017a).

Co-ligation of CD150 with CD180 results in attenuation of the phosphorylation of both the AKT and p38MAPK pathways in CLL cells (Fig. 6). Notably, co-activation of CD40 and CD150 does not attenuate AKT or p38MAPK phosphorylation, indicating that usage of these pathways is receptor specific. It is not clear, however, if the reduction in the activation of these pathways is important for modulating CLL cell survival, as seen previously with CD180-mediated dichotomous intracellular signaling (Porakishvili et al. 2015).

Interestingly, CD150-mediated AKT activation in CLL cells is accompanied with an increase in the phosphorylation of the transcription factors FoxO1 and FoxO3a. Simultaneous ligation of CD150 with CD180 with mAbs results in a decrease in *FOXO* transcription (Gordiienko et al. 2017a), suggesting that together they promote a pro-survival phenotype. Independent ligation of CD150 or CD180 leads to upregulation of *BCL6* mRNA (Gordiienko et al. 2017b). Since, in general, BCL6 is an anti-apoptotic protein and a transcriptional regulator of *TP53* (Phan and Dalla-Favera 2004), this may represent one modality that contributes to a pro-survival phenotype following anti-CD180 or anti-CD150 treatment of  CLL cells. Ligation of CD180 or CD150, individually or together, leads to a strong down regulation of IRF4 and upregulation of IRF8 and PU.1 (Gordiienko et al. 2017b), further supporting the idea that CD180 and CD150 can share intracellular signaling and transcriptomic pathways. IRF4 is a transcription factor important in regulating plasma cell differentiation (Shapiro-Shelef and Calame 2005) and IRF8 and PU.1 are involved in the activation of follicular and germinal centre B cells (Wang et al. 2019) and the development of pre-B cells (Pang et al. 2016). This could be important to CLL cells present in proliferation centres in lymph nodes (Edwards et al. 2021), indicating that activation and proliferation of CLL cells might result from a concordant ligation of CD180 and CD150.

### Multiple myeloma

Whereas bone marrow plasma cells do not normally express CD180, multiple myeloma (MM) cells do, and this increased expression is promoted by the hypoxic bone marrow microenvironment (Kikuchi et al. 2018; Egli et al. 2015). Both LPS challenge and anti-CD180 mAb treatment promoted the growth of MM cells, in vitro (Kikuchi et al. 2018). Moreover, LPS appeared to induce clonal expansion of CD180-positive MM cells and notably, knockdown of CD180 reduced the LPS responsiveness of MM cells further indicating the role of CD810 in the detection of LPS by TLR4. It is likely that these two receptors operate together to promote MM cell growth (Kikuchi et al. 2018). Since CD19 can enhance LPS-mediated growth by the CD180/MD1 complex, it might also play a major role in regulating signaling via CD180 (Yazawa et al. 2003). However, it should be noted that CD19 is only expressed by a very small proportion of MM cells as measured by flow cytometry (Tembhare et al. 2014). Although, super-resolution microscopy has revealed that MM cells do express CD19, albeit at very low densities (Nerreter et al. 2019).

## CD180 in autoimmune disorders and other inflammatory pathologies

CD180 has been extensively studied within the context of autoimmune disease and other non-malignant inflammatory disorders where it appears to play a pathophysiological role.

### CD180 in systemic lupus erythematosus (SLE)

Systemic lupus erythematosus (SLE) is a multisystem disease characterized by immune dysregulation and production of autoantibodies, typically against double stranded DNA (dsDNA) (Wang and Xia 2019). A high proportion of CD180-negative B cells are found in SLE patients (15 ± 11.7%) compared with healthy controls (1.7 ± 1.1%) (Koarada et al. 1999). Moreover, clinical observations show that the higher proportions of CD180-negative B cells associate with active disease (Koarada et al. 1999). Furthermore, increased proportions of CD180-negative B cells correlate with increased renal legions in murine lupus-like nephritis models (Fujita et al. 2012) and CD180-negative SLE B cells are primarily responsible for the production of anti-double stranded DNA autoantibodies (Koarada et al. 1999; Kikuchi et al. 2002). Interestingly, CD180-negative B cells from patients with SLE also exhibit increased production of antibodies to dsDNA in response to IL-6 or challenge with SAC, whereas CD180-positive B cells are unresponsive to such stimuli (Kikuchi et al. 2002).

Mechanistically, the elevated autoimmune responses could relate to B cell activation since CD180-negative SLE B cells overexpress B cell maturation antigen (BCMA) (Koarada et al. 2010). Additionally, sCD40L trimers, which are elevated in SLE, reduce the levels of viable CD180-negative (and CD180-positive) SLE B cells, and this effect is reversed by treatment of B cells with the BCMA ligand APRIL (a proliferation-inducing ligand) (Koarada et al. 2010). Therefore, the preferential expression of BCMA by CD180-negative B cells could promote plasma cell differentiation, thereby perpetuating a more severe disease phenotype.

TLRs contribute to the pathophysiology of SLE. As discussed above, CD180 can negatively regulate TLR activity. Therefore, loss of CD180 could enable TLR-mediated cellular activation and inflammation, thus promoting SLE progression. In support of this, Yang et al. showed that CD180 activation inhibits expression of TLR7 and TLR9 in murine macrophages and DCs; notably, patients with SLE also have increased frequencies of CD180-negative macrophages and DCs (Yang et al. 2018a). Given that TLR7 and TLR9 are expressed by B cells, and CD180 can interact with the downstream pathways of these receptors in B cells, it will be important to study the functional role of TLR7 and TLR9 in CD180-positive and CD180-negative SLE B cells.

Together, these data point towards a protective effect for CD180 in SLE. Loss of CD180 on APCs in SLE correlates with disease activity, and functional studies in mice demonstrate that activation of CD180 can help to ameliorate SLE disease activity (Yang et al. 2018a). Gaining a deeper understanding into the function of CD180 in SLE, and how this is differentially downregulated may aide in the development of new treatment approaches or improve the accuracy of predicting disease behaviour.

### CD180 in rheumatoid arthritis (RA)

To date, the authors have found no publications of studies on human rheumatoid arthritis (RA) disease. However, there is a study where the murine collagen-induced arthritis (CIA) model has been used to study the role CD180 in RA. CD180^−/−^ mice demonstrate more severe CIA with an earlier onset, compared to wild type mice (Tada et al. 2008). Further, CD180^−/−^ mice more robustly produce INF-γ and TNF-α, inflammatory mediators that play important roles in the pathophysiology of RA (Williams et al. 1992; Manoury-Schwartz et al. 1997).

### Kawasaki disease

Kawasaki disease (KD) affects infants and children, leading to inflammatory changes of blood vessels which is often accompanied by fever and lymph node swelling. Patients also develop inflammation of cardiac tissues and valves, and, in some cases, aneurysms or thrombi are formed. The causes of KD are unknown, although the onset of the disease appears related to infection as supported by epidemiological observations of endemic KD (Agarwal and Agrawal 2017).

There is an indication that children with KD are characterized with expansion of peripheral blood B cells with CD180-positive phenotype, compared to control samples (Imayoshi et al. 2006). Notably, patients with viral infections similarly exhibit upregulated proportions of CD180-positive B cells, although to a lesser degree, further indicating a link between viral infection and KD. From the limited number of observations, the importance of CD180 expression on expanding population of B cell was unclear. However, CD180 expression was upregulated on KD B cells compared to normal controls and those with viral infections (Imayoshi et al. 2006).

It is known that B cells are activated in KD, producing antibodies, perhaps in response to viral or bacterial infection. Given the preferential expression of CD180 by memory B cells and the involvement of CD180 in B cell activation and immunoglobulin production, it is possible that CD180 plays a role in the development and pathophysiology of KD. CD180-overexpressing B cells in KD could therefore represent a subset of activated B cells.

Differences in CD180 might also modulate the function of other receptors in B cells in KD. For example, given the known functional interactions with classical members of the TLR family, CD180 could modulate TLR function which could, in turn, lead to the production of pro-inflammatory cytokines and perpetuation of the KD phenotype. Interestingly, CD150 is also upregulated by memory B cells (Good et al. 2009), and therefore upregulated expression of CD180 by KD B cells might also affect CD150 function, as mentioned above. Phenotypic and functional analysis of B cells as well as macrophages and DCs may shed further light on the exact role of CD180 in KD pathophysiology.

Recently, SARS-CoV-19 infection has been implicated as an etiological factor in the initiation of KD or a KD-like syndrome. Observational studies indicate significant increases in Kawasaki-like disease in areas with high prevalence of SARS-CoV-19 infection, suggesting a role for the novel Corona virus in the presentation of KD (Verdoni et al. 2020; Sancho-Shimizu et al. 2021). To our knowledge, there are no data on CD180 expression in SARS-COV-19 or related systemic disorders. Given the upregulation of CD180 in both KD and viral infection (Imayoshi et al. 2006), analysing changes in CD180 expression may be useful for predicting which individuals may present with Kawasaki-like disease or systemic inflammation in SARS-CoV-2 infection.

### CD180 in autoimmune disorders of the central nervous system (CNS)

Data from anti-CD20 B cell studies have suggested that B cells play a role in the pathogenesis of neuromyelitis optica spectrum disorder (NMOSD) and multiple sclerosis (MS). Hayden et al. showed that CD180 expression was exclusively decreased in the CD19^+^CD27^+^IgD^+^ non-switched (NS) memory B cells in both NMOSD and MS compared to healthy controls (Hayden et al. 2021). Furthermore, the same group showed that there were increased titres of anti-citrate synthase (anti-CS) autoantibodies in patients with autoimmune disease (Czömpöly et al. 2006). Anti-CS IgM autoantibody serum level was also lower in both NMOSD and MS suggesting that reduced CD180 expression of NS B cells could contribute to the deficient IgM autoantibody production in these disorders.

### Other autoimmune diseases and chronic inflammatory conditions

Differential expression of CD180 has also been reported in other autoimmune conditions (Table 2). Kikuchi et al. showed that patients with dermatomyositis present with increased proportions of CD180-negative B cells compared to patients with polymyositis and normal controls (Kikuchi et al. 2001). Some patients with IgG4-related disease (IgG4-RD) also present with CD180-negative B cells (Koarada et al. 2013) and loss of CD180 corresponded with increased disease activity in IgG4-RD. These studies are, however, limited by the fact they are case reports.

**Table 2:** The differential expression of CD180 in various autoimmune and inflammatory pathologies

Disease	Cell or tissue	Methodology	CD180 expression relative to healthy controls	References
Neuromyelitis optica spectrum disorder and multiple sclerosis	B cells	FC	↓	(Hayden et al. 2021)
Diffuse cutaneous systemic sclerosis	B cells	qPCR, FC	↓	(Simon et al. 2021; Koarada et al. 2001; Erdo-Bonyar et al. 2019)
Dermatomyositis	B cells	FC	↓	(Kikuchi et al. 2001; Koarada et al. 2001)
Kawasaki disease	B cells	FC	↑	(Imayoshi et al. 2006)
Sjogren’s syndrome	B cells	FC	↓	(Koarada et al. 2001; Kikuchi et al. 2008)
IgG4-related disease	B cells	FC	↓	(Koarada et al. 2013)

*FC* flow cytometry, *qPCR* quantitative polymerase chain reaction

### CD180 in cardiac injury and atherosclerosis

TLRs play an important role in the pathophysiology of cardiovascular disease, and CD180 has also been investigated within the context of cardiovascular pathologies. WT mice develop significantly worse cardiac damage following myocardial infarction compared to mice where CD180 was overexpressed in murine cardiac tissue by adenovirus transfection, suggesting that CD180 plays a protective role in cardiac injury (Louwe et al. 2014). Building on this work, it was shown that CD180 overexpression can ameliorate cardiomyocyte apoptosis via inhibition of TLR4 signaling and p38MAPK phosphorylation signalin ischemia/reperfusion injury rat models (Li et al. 2016; Yang et al. 2015). Moreover, in cardiomyocytes, CD180 was able to effectively reduce TLR4-mediated production of pro-inflammatory IL-6 and TNF-α in response to ischemia (Li et al. 2016).

The protective effect of CD180 in ischemia could be dampened through the interference of microRNAs, which are involved heavily in cardiovascular pathologies. Specifically, miR-327, which is preferentially expressed in myocardium, downregulates CD180 and thus promotes proinflammatory TLR4 activation (Yang et al. 2018b). However,  miR-383 was shown to promote myocardial health in ischemia and reperfusion injuries in a CD180-dependent manner (Guo et al. 2021). CD180 therefore is a potential therapeutic target in cardiac injury, perhaps through the indirect amelioration of TLR4-mediated inflammation (Yang et al. 2019). Furthermore, a recent study also implicated CD180-mediated inactivation of TLR2 as a potential pathway which promotes cardiac repair following myocardial infarction (Huang et al. 2020).

The pathogenesis of atherosclerosis is largely immunological in nature. B cells play a role in atherosclerotic plaque formation by producing pro-inflammatory cytokines and through B cell activation by neo-antigens (Sage et al. 2019). Understanding changes in B cell function is therefore important for elucidating mechanisms of atherosclerotic plaque development. CD180 is expressed on murine vascular smooth muscle cells, along with TLR4, indicating a more direct role for CD180 in cardiovascular health (Karper et al. 2013). Neointima formation is a common feature of atherosclerotic plaque development and is characterized by an increase in vascular smooth muscle cells within a vessel which can promote atherosclerotic plaque development. Loss of CD180 is associated with increased formation of neointima scar tissue in mice with artery cuffs that are used to induce vascular damage (Karper et al. 2013), suggesting a protective role for CD180. Furthermore, local addition of LPS increased neointima formation, although the increase was more dramatic in CD180^−/−^ mice compared to wild-type animals. Later, however, somewhat conflicting evidence emerged: in low density lipoprotein receptor (LDLr)^−/−^ CD180^−/−^ mice, there is marked reduction in plaque formation compared to LDLr^−/−^ animals (Karper et al. 2013). Wezel et al. also demonstrated that CD180-negative mice showed reduced plaque formation, as well as reduced monocyte migration (Wezel et al. 2015). This was accounted for by a greater reduction in the chemokine ligand 2 (CCL2) in CD180^−/−^ mice compared to those with intact CD180. These two studies suggest that CD180 expression in fact promotes plaque formation. Consistently, Wezel et al. later showed that CD180 deficient mice had an increased number of unstable lesions in murine vein graft models, which was attributed to increased CCL2 production and thus mast cell recruitment (Wezel et al. 2016).

Together, these data suggest that whilst CD180 is associated with reduced neointima formation, thus decreasing the risk of plaque development, paradoxically CD180 simultaneously promotes atherosclerotic plaque development. Understanding the mechanistic role of CD180 in modulating the immune cells which promote atherosclerotic plaque development may provide a rationale for CD180 targeted therapies.

## The current perspective: a model of CD180 in disease

CD180 is highly expressed by APCs from healthy individuals where it plays a role in cell activation and modulation of immunoglobulin production. Moreover, it interacts with various other receptors to regulate the action of classical TLRs. However, there is differential expression and function of CD180 in disease.

The literature promotes the overarching idea that CD180 is often differentially downregulated or lost in disease. Notably, CD180 tends to be downregulated in hematological malignancies, autoimmune diseases, and some inflammatory conditions which therefore suggests that CD180 promotes health. There are, however, some conditions where CD180 expression becomes upregulated and this corresponds with the aetiology of the disease, rather than being directly disease related. For example, the increased upregulation of CD180 in KD could be due to the causative infectious agent, rather than a manifestation of KD itself. The upregulation in the expression of CD180 in KD may serve as a physiological response, perhaps to dampen hyperactive immune responses to bacteria or viruses. This concept is consistent with findings which show that B cells respond to stimulation with synthetic RNA by upregulating CD180 expression (Imayoshi et al. 2006).

The negative effect which CD180 has on classical TLRs suggests that CD180 may become upregulated in response to infection to aid prevention of hyperinflammation. Perhaps the most notable interaction is with TLR4, where CD180 negatively regulates TLR4-mediated responses to LPS (Divanovic et al. 2005). Therefore, the upregulation of CD180 could serve to prevent hyperinflammation during infection, thereby preventing sepsis. Notably, there is significant upregulation of CD180 by some subsets of immune cells in LPS-challenged mice (Dong et al. 2019). Similarly, in *Mycobacterium tuberculosis* infection, human macrophages increase their expression of CD180 (Blumenthal et al. 2009).

CD180 does not, however, always conform to these behaviours. For example, CD180 expression by murine macrophages does not increase in response to *Mycobacterium avium* infection (Blumenthal et al. 2005). Furthermore, in cardiovascular pathologies, CD180 appears to play an antagonistic role to promote atherosclerotic plaque formation. Therefore, it is not entirely possible to conclude a defined function for CD180 that unifies all the current data. Rather, it appears that the immunopathology of CD180 is contextual and is dependent on (1) the cell by which CD180 is expressed and (2) the disease in which CD180 is functioning. The molecular architecture of CD180 supports this idea; given its short intracytoplasmic tail, CD180 likely requires partner receptors to initiate intracellular signaling. The unique profile of receptor expression by different cell types, coupled with the differential expression of CD180 (and other TLRs) on APCs in pathology, could serve to modulate the behaviour of CD180 and thus explain the divergent properties observed in studies of CD180 function.

### CD180 as a prognostic biomarker

It is now well documented that M-CLL is associated with superior prognosis in CLL compared to U-CLL (Agathangelidis et al. 2022). Now data are accumulating to suggest that CD180 could be useful as a prognostic biomarker in different diseases. Levels of *CD180* mRNA are upregulated in patients with osteosarcoma with superior outcomes (Chen et al. 2020). At the protein level, proportions of CD180-negatve B cells correspond with SLE disease activity (Koarada et al. 1999). Similarly, we have found an association with CD180 expression and disease outcomes in CLL. We discovered that increased CD180 expression in LNs was associated with early-stage disease and superior overall survival (Edwards et al. 2021). This is consistent with earlier observations that CD180 is more highly expressed in M-CLL (Porakishvili et al. 2005) and positively correlates with CD150 (Gordiienko et al. 2017a), both of which associate with a positive prognostic picture in CLL (Bologna et al. 2016). Together, these findings suggest that prediction of disease severity and progression could be benefitted through study of CD180 expression.

The diverse molecular nature of CD180 signaling could also be utilized to provide better resolution in CLL disease prediction or even therapy response. Among responder CD180-positive CLL cases, the dichotomous activation of AKT or p38MAPK in CLL cells following CD180 ligation, respectively, corresponds with survival or apoptosis (Porakishvili et al. 2015). Should this reflect an in vivo occurrence, CLL cases that signal via AKT could represent a subset of patients with increased tumor proliferation and perhaps a more aggressive disease course. Alternatively, p38MAPK-s cases could represent a subset of patients with less aggressive disease. CD180-negative cases and CD180-positive non-responders are also unresponsive to cumulative anti-CD40 and IL-4 simulation (Porakishvili et al. 2011) or IgM ligation (unpublished observations) and therefore appear to represent a subset of CLL cases with anergic leukemic cells: a phenotype typically associated with an indolent disease course (Apollonio et al. 2013).

Understanding how CD180 is co-expressed and interacts with other receptors could also further improve the prognostic utility of CD180. A significant proportion of AKT-S CLL cases (70%) responded to anti-IgM by activation of the BTK/PI3K/AKT axis, whilst only a small proportion of p38MAPK-S cases responded to anti-IgM by increasing the phosphorylation of p38MAPK (Porakishvili et al. 2015). This presents the idea that AKT-S cases are much more receptive to IgM engagement compared to p38MAPK-S cases which may help to explain why this subset of patients readily recruits BCR machinery, potentially helping to promote a survival phenotype by CLL cells. However, it is not clear if these differences were due to differential expression of ZAP-70 and CD38 in this study. The capacity of CD180 to rewire IgM-mediated pro-survival signaling to promote apoptosis (Porakishvili et al. 2015) and the tendency of CD180 to cooperate with IgD to activate p38MAPK indicates that studies of the different BCR isoforms may be useful for defining the prognostic capacity of CD180 (Porakishvili et al. 2017).

Understanding how CD180 activation and coreceptor interactions affect the CLL signalosome could also be exploited for prediction of therapy responses. Should CLL cells differentially downregulate the use of signaling axes which depend on BKT or PI3K, it is plausible that these patients may be resistant to the BTK and PI3K protein kinase inhibitors ibrutinib or idelalisib. Therefore, since CD180 can modulate the phosphorylation and utilization of BTK and PI3K, it may be a strong candidate to predict protein kinase inhibitor responsiveness.

Similarly, there are data to suggest that CD180 may also be useful as a biomarker to indicate pharmacodynamic responses to bromodomain (BRD) inhibitors, which have shown pre-clinical efficacy in hematological malignancies such as MDS and AML. When a range of hematological cancer cell lines, including ALL, MM, and DLBCL types, were treated with AZD5153, a novel BRD4 inhibitor, all demonstrated considerable reduction in *CD180* mRNA levels (Yeh et al. 2017). Significant and robust CD180 mRNA down regulation was also observed in ALL murine xenograft models, and in peripheral blood in vitro studies of CLL and MM patients, as well as healthy controls. Whilst further validation is required in larger, prospective cohorts and with different BRD4 inhibitors, the consistency in the reduction of *CD180* mRNA across the different experimental set-ups is striking. In addition to *CD180, chemokine receptor type 2 (CCR2)* mRNA levels were downregulated in response to AZD5153 treatment, thus BRD4 inhibition may modulate the transmigration of tumor cells within the microenvironment (Macanas-Pirard et al. 2017). Together, these data suggest that *CD180* mRNA levels may be a relevant pharmacodynamic biomarker for BRD4 inhibitors in hematological malignancies. Furthermore, recent studies advocate the rationale of the use of BRD inhibitors in CLL (Ozer et al. 2018; Jiang et al. 2020). BRD inhibitors may also increase the sensitivity to venetoclax, and thus could be employed as an adjuvant therapy in CLL (Carrà et al. 2020).

Taken together, these data suggest that through studying CD180 expression and activation, it may be possible to better predict and explain the observed heterogeneity in CLL tumor aggressiveness, progression, and therapy responsiveness. Moreover, further insights into the behaviour of CLL cells could be achieved by studying the expression and co-engagement of CD180 with other surface receptors, namely the IgD and IgM BCR isoforms. The true utility of CD180 as a biomarker would, however, need to be fortified by relating signaling activity with longitudinal clinical data on disease progression, survival, and therapy responses. The applicability of these data to other hematological malignancies is also yet to be established. However, it is known that CD180 expression is modulated in various B cell malignancies, other than CLL. Therefore, it is possible that CD180 may also be of value for predicting outcomes in these cancers.

### CD180 as a therapeutic target

Although there are no clinical trials reported to date, the data suggest that CD180 could be a potential therapeutic target. Our group showed that pre-engagement of CD180 with anti-CD180 followed by treatment with anti-IgM in AKT-s CLL cells significantly decreases levels of p-AKT, increases p38MAPK, and increases the number of apoptotic cells (Porakishvili et al. 2015). An earlier study in mice also demonstrated that pre-treatment with anti-CD180 can program B cells to undergo apoptosis following IgM ligation (Yamashita et al. 1996).

These findings are significant as they demonstrate that CD180 may be a targetable molecule, given that it can reprogram certain populations of CLL clones to undergo apoptosis following BCR engagement. Since CLL and other malignant B cells depend heavily on the BCR for survival signals and evasion of apoptosis, priming of malignant B cells to undergo apoptosis via BCR engagement following pre-treatment with a molecule to activate CD180 is an attractive treatment strategy. Notably, the rewiring phenomenon did not occur in control B cells pre-treated with anti-CD180 and then anti-IgM (Porakishvili et al. 2015). Therefore, engagement of CD180 may specifically promote the apoptosis of CLL cells, leaving healthy B cells unaffected.

If the same program of dichotomous signaling is present in other types of malignant cells, then the rationale for this treatment approach could be extended. Further, targeting of malignancies which express CD180 highly on the cell surface could also help to improve the efficacy of this potential modality of treatment. The augmentation of BCR signaling to facilitate apoptosis via BCR engagement is also a particularly attractive strategy for the treatment of U-CLL. This is because CLL cells with unmutated *IGHV* genes are in a heightened state of BCR activation (Burger and Chiorazzi 2013) which could assist CLL clones in rewiring their signals to promote apoptosis more readily. This is supported by an earlier study which demonstrated that U-CLL cells respond more readily to IgM cross-linking (Lanham et al. 2003). It is first required, however, to accurately stratify patients by the tumor’s capacity to rewire IgM signals to promote apoptosis. This approach would obviously not be applicable to cases of CLL or other B cell cancers where both CD180 and IgM are not expressed. Notably, however, CD180 tends to be downregulated in U-CLL, compared to M-CLL (as discussed previously). Thus, such an approach may be useful only in a fraction of U-CLL cases.

It has also been suggested that rather than engaging CD180, utilizing CD180 expression on B cell subsets could be a way to exploit CD180 for therapy. Koarada and Tada suggested that targeting of the autoantibody producing CD180-negative B cells could be an effective treatment modality of SLE (Koarada and Tada 2012). Targeting of CD180-negative B cell populations may be possible since CD180-negative B cell subsets have an increased expression of certain antigens, such as BCMA in SLE (Koarada et al. 2010).

Since it is known that CD180 can negatively regulate both TLR4 and TLR2 signaling (Divanovic et al. 2005), an alternative treatment approach in inflammatory disorders may be to increase CD180 expression which could in turn, help to downregulate proinflammatory cytokines and reduce tissue damage. One approach could be to silence microRNAs, which negatively regulate CD180 expression. Multiple microRNAs, including miR-141-p, miR-327, and miR-383, can interfere with CD180 expression and inhibition of these microRNAs can ameliorate cardiac tissue damage in ischemia models (Yang et al. 2018b; Qin et al. 2019; Guo et al. 2021). This modality therefore might represent a target for therapeutic intervention.

## Conclusions

In physiology, CD180 plays roles in B cell activation and modulation of TLR responses. CD180 provides cellular stimulation and interacts with other receptors to modulate APC function in a heterogenous way. It appears that the heterogeneity in function is dependent on the availability of other receptors. This is likely due to the molecular nature of CD180. Its short cytoplasmic tail and lack of functional intracellular signaling domain mean that CD180 requires signaling partners to propagate extrinsic cues. Which ligands initiate CD180 signaling, in vivo*,* remain unknown, and their discovery will aide in a better understanding of the function of CD180 in physiology. It appears, however, that even as an orphan receptor, CD180 functions to modulate other receptors, particularly TLR4.

CD180 expression becomes either upregulated or downregulated on B cells in diseases such as SLE, cardiac pathologies and B cell malignancies. The aberrant expression of CD180 in disease is also accompanied by functional differences, particularly in CLL where the receptor has apparently unique signaling properties which can powerfully regulate CLL cell behaviour and the action of the BCR.

Expression and functional studies are accompanied by data which indicate a role for CD180 in disease prognostication, with increased CD180 expression being generally associated with a less severe disease phenotype, although this is not always the case. Further studies which define CD180 signaling behaviour at a greater resolution will aid in developing CD180 as a prognostic biomarker and may help to highlight potential therapeutic avenues, particularly in CLL, SLE and cardiovascular pathologies.

## Data Availability

The datasets used and/or analysed during the current study are available from the corresponding author on reasonable request.

## Abbreviations

BCR: B cell receptor
Ag: Antigen
AKT: Protein kinase B
AML: Acute myeloid leukemia
APC: Antigen presenting cells
ATRA: All-*trans* retinoic acid
BAD: B cellleukemia/lymphoma 2 protein (BCL2)-associated agonist of cell death
BAFF: B cell activating factor
BCL-_XL_: B cell lymphoma-extra large
BCMA: B cell maturation antigen
BLC6: B cell leukemia/lymphoma 2 protein
BTK: Bruton's tyrosine kinase
CCL2: Chemokine ligand 2
CCL5: Chemokine ligand 5
CIA: Collagen induced arthritis model
CLL: Chronic lymphocytic leukemia
DAMP: Damage-associated molecular pattern
DCs: Dendritic cells
FC: Flow cytometry
FITC: Fluorescein isothiocyanate
FO: Follicular
FL: Follicular lymphoma
HEK: Human embryonic kidney cells
Ig: Immunoglobulin
*IGVH*: *Immunoglobulin Heavy Chain Gene*
IL-4: Interleukin-4
IL-6: Interleukin-6
IL-7: Interleukin-7
IRF8: Interferon regulatory factor 8
KD: Kawasaki disease
KO: Knockout
LDLr: Low density lipopolysaccharide receptor
LPS: Lipopolysaccharide
Lyn: Tyrosine-protein kinase Lyn
mAb: Monoclonal antibody
MAMP: Microbe associated molecular patterns
MCL: Mantle cell lymphoma
Mcl-1: Induced myeloid leukemia cell differentiation protein Mcl-1
M-CLL: Mutated chronic lymphocytic leukemia
MD-1: Myeloid differentiation 1
MD-2: Myeloid differentiation 2
MDS: Myelodysplastic syndrome
MHC II: Major histocompatibility complex II
miRNA: MicroRNA
MS: Multiple sclerosis
MM: Multiple myeloma
MyD88: Myeloid differentiation primary-response protein 88
MZ: Marginal zone
MZL: Marginal zone lymphoma
NF-κB: Nuclear factor kappa-light-chain-enhancer of activated B cells
NMOSD: Neuromyelitis optica spectrum disorder
NS: Non-switched; non-signaler
OxPAPC: Oxidised 1-palmitoyl-2-arachidonoyl-sn-glycero-3-phosphorylcholine
p38MAPK: P38 mitogen-activated protein kinase
Pam3CSK4: Synthetic bacterial lipopeptide Pam3-Cys-Ser-Lys4
PE: Phycoerythrin
PI3K: Phosphatidylinositol-3 kinase
PU.1: Transcription factor PU
qPCR: Quantitative polymerase chain reaction
RA: Rheumatoid arthritis
RFI: Relative fluorescent intensity
SAC: Staphylococcus aureus Cowan
SLE: Systemic lupus erythematosus
SYK: Spleen associated tyrosine kinase
TIR: toll-like/interleukin-1 receptor
TIRAP: Toll/Interleukin 1-receptor adaptor protein
TLR: Toll-like receptor
TNF-α: Tumor necrosis factor-alpha
*TP53*: Tumor protein 53
TRAM: Toll/IL-1R domain-containing adaptor-inducing interferon-BETA related adaptor molecule
TRIF: Toll-interleukin-1 receptor homology domain (TIR)-domain-containing adapter-inducing interferon-BETA
U-CLL: Unmutated chronic lymphocytic leukemia
ULK1: Unc-51 like autophagy activating kinase 1
WT: Wild-type
